# Assessment of knowledge, attitude, and practice regarding medical waste management among operation room personnel in a tertiary hospital

**DOI:** 10.1097/MS9.0000000000002212

**Published:** 2024-07-17

**Authors:** Yosef Belay Bizuneh, Yonas Admasu Ferede, Yophtahe Woldegerima Berhe, Wudie Mekonnen Alemu, Tadael Gudayu Zeleke

**Affiliations:** Department of Anaesthesia, College of Medicine and Health Sciences, University of Gondar, Gondar, Ethiopia

**Keywords:** attitude, knowledge, medical waste, practice, waste management, waste segregation

## Abstract

**Introduction:**

Medical waste management (MWM) is of concern to the medical and surgical communities in the hospital. Adequate knowledge, attitude, and practice regarding the management of healthcare waste are important for the proper handling and disposal by physicians and healthcare workers.

**Objective:**

The aim of this study was an assessment of knowledge, attitude, and practices regarding medical waste management among operation room personnel in a tertiary hospital.

**Methods:**

This study was a descriptive cross-sectional survey conducted at a single-center government Comprehensive Specialized Hospital operation room from 1–30 September 2022. All specialty operation room healthcare personnel consenting to cooperate and participate were included in the study. The data were collected using a self-administered questionnaire. The collected data were analyzed using SPSS. The results were presented in texts, tables and graphs.

**Results:**

From 130 operation room personnel, the results revealed that the majority of the total respondents were male (83.1%). Only 30 (23.1%) of the respondents were involved in training, whereas 100 (76.9%) of the study participants have not participated in training. It was found that 86 (66.2%) of the respondents had good knowledge, 113 (86.9%) of the respondents had a good attitude, and 27 (20.8%) of the respondents had good practice.

**Conclusion and recommendation:**

The authors’ study reveals that the majority of study participants have a moderate level of knowledge, a good level of attitude, and poor levels of practice, according to Bloom’s cut-off point. The institution should more fully implement the training for operation room personnel to improve their level of knowledge and practice of medical waste management.

## Introduction

Highlights

- Medical waste is regulated medical waste.
- Medical waste management is of concern to the medical and surgical communities.
- A significant source of medical waste is produced in the operating room.

A significant source of medical waste is produced in the operating room (OR). Studies note that operation rooms produce 20–33% of all waste in the hospital setting^1–3^. Although medical waste is produced at a higher rate, the proper management of medical waste is suboptimal^4^. The greatest challenge with proper disposal of medical waste management is the correct segregation of waste^5^.

Although 90% of all operation room waste is non-hazardous and potentially recyclable, 30–90% of this waste is incorrectly disposed of as hazardous^1,6^. Medical waste is consistently and inappropriately segregated, leading to environmental pollution, misuse of resources, and subsequent increased costs for medical facilities^1–4^.

Medical waste is divided into four types of waste streams: solid waste, regulated medical waste, pharmaceutical waste, and recyclable waste^4,6^. Solid waste is any item that is lightly contaminated or non-recyclable^7–9^. Regulated medical waste is any waste that is saturated with bodily fluids or blood and can be further divided into sharps and red bag waste^10–12^. Pharmaceutical waste is any form of medication that is not used, partially, used, or contaminated^13^. Recyclable waste is clean paper, plastic, or empty unbroken glass^14^. The opportunity for recyclable waste in the OR has a tremendous impact on the environment^15^.

Many factors, such as socio-economic status, regulatory policies, level of education, available resources, treatment technologies, and capability of managing best practices, contribute to the variability of medical waste management^16^.

Institutions typically have established waste management systems. However, in many institutions, waste is still being improperly segregated^10,17,18^. Roughly 59–70% of regulated medical waste (RMW) contains potentially recyclable or general waste^19^. Educational initiatives, including online-based modules, in-person sessions, and audio-visuals, have been shown to improve knowledge of terminologies, classifications, and segregation^20–23^. These initiatives showed a 30% increase in segregation at the source, a 41–59% decrease in regulated medical waste (RMW), a 77% reduction in non-regulated items, and a 19% increase in recyclable waste^22,24^. The aim of this study was an assessment of knowledge, attitude, and practices regarding medical waste management among operation room personnel in a tertiary hospital.

## Methods

### Research design, setting, and study population

This study was a descriptive cross-sectional survey conducted at a single-center government Comprehensive Specialized Hospital operation room, from 1–30 September 2022. Data were collected as a survey consecutively in all specialty operation room healthcare personnel. The personnel included were surgeons, obstetricians and gynecologists, residents, anesthetists, nurses, and midwifery staff. Personnel who could not understand and follow instructions and those not willing to participate were excluded.

The University Hospital is a comprehensive, specialized, and teaching hospital that provides health services for more than five million people in the catchment area. The hospital has 12 major operation rooms. According to the Hospital Planning and Program Coordination Office report, 4125 patients were operated on under anesthesia per year in 2020.

The Research Registry number was stated as 10082, in accordance with the Declaration of Helsinki,2013^25^. This study has been reported in line with the STROCSS criteria^26^.

### Sample size, sampling procedure, data collection tools, and methods of measurement

One hundred thirty operation room personnel were involved in the cross-sectional survey. Data were collected as a survey consecutively in all specialty operation room healthcare personnel. A questionnaire was used in this study to determine the knowledge, attitude, and practice of medical waste management among healthcare personnel. The questionnaire contained both open-ended and closed-ended items and was divided into five parts, as follows: part A: socio-demographic characteristics of the respondents; part B: general information from respondents regarding MWM at the hospital; part C: knowledge of respondents about MWM; part D: attitudes of respondents towards MWM; and part E: practice of respondents in respect of MWM.

In each part, the items were designed to draw out information about the respondents’ Knowledge, Attitude and Practice (KAP) relating to four aspects of medical waste: segregation, collection, transportation, and final disposal. Details of the items in the questionnaire used to obtain information about those four aspects were as follows:

Knowledge of MWM was assessed using 14 items, such as follows: medical waste is a vaccine container; medical waste is material contaminated with body fluids; waste generated from healthcare activities is medical waste; medical waste should not be mixed with general waste; medical waste should be segregated immediately; and the color coding for medical waste is red. The knowledge items were scored as either “1” or “0” for the correct or incorrect response, respectively. The total knowledge score for each respondent could range from a minimum of 0 to a maximum of 14. The overall knowledge was categorized, using Bloom’s cut-off point, as good if the score was between 80 and 100%, moderate if the score was between 60 and 79%, and poor if the score was less than 60%.

Attitude towards MWM was assessed using 10 items, such as: general waste management and medical waste management are different; medical waste segregation is important; medical waste must be collected more carefully; and medical waste management in your hospital is proper. A 3-point Likert scale was used to respond to the items in the attitude section, where “agree,” “disagree,” and undecided were scored as 2, 1, and 0, respectively. The total attitude score for each respondent could range from a minimum of 0 to a maximum of 20. The overall attitude was categorized, using Bloom’s cut-off point, as good if the score was between 80 and 100%, moderate if the score was between 60 and 79%, and poor if the score was less than 60%.

Practice in respect of MWM was assessed with 11 questions, such as: How often do you separate medical waste from general waste? Do you not put sharp medical waste into a red plastic bag? Do you put sharp medical waste in a hard container? And do you wear rubber gloves when picking up trash that falls on the ground? The participants were asked to respond to these questions based on a 3-point Likert scale, where “always,” “sometimes,” and “never” were scored as 2, 1, and 0, respectively. The total practice score for each respondent could range from a minimum of 0 to a maximum of 22. The overall practice was categorized, using Bloom’s cut-off point, as good if the score was between 80 and 100%, moderate if the score was between 60 and 79%, and poor if the score was less than 60%.

### Data analysis

The data were collected using a self-administered questionnaire. The questionnaire was pretested to ascertain ease of understanding and to determine if it was worded to elicit all the materials of interest for this research study. Therefore, this process was concerned with assessing the content validity of the questionnaire. Participants for the pretesting stage were drawn from the heads of departments at the study hospital, which included doctors and nurse practitioners. The pretesting of the questionnaire was conducted at the same hospital as the study; however, those involved in the pretesting phase were not allowed to participate in the actual study. Findings from this process showed that all respondents were satisfied and that the questionnaire was adequate for the purpose of the study. The final version of the questionnaire consisted of the following five sections: Socio-demographic status, general information from respondents regarding waste management, knowledge of waste management, attitudes toward waste management, and practice of waste management. The collected data were analyzed using SPSS. The results are presented in texts, tables and graphs.

### Operational definitions

The overall knowledge was categorized, using Bloom’s cut-off point, as good if the score was between 80 and 100%, moderate if the score was between 60 and 79%, and poor if the score was less than 60%^27–29^.

The overall attitude was categorized, using Bloom’s cut-off point, as good if the score was between 80 and 100%, moderate if the score was between 60 and 79%, and poor if the score was less than 60%^27–29^.

The overall practice score will be categorized using the same Bloom’s cut-off point, as good if the score is between 80 and 100%, moderate if the score is between 60 and 79%, and poor if the score is less than 60%^27–29^.

## Results

Part A: Socio-demographic characteristics of the respondents: From 130 operation room personnel, the results revealed that the majority of the total respondents were male (83.1%), with the most common age range being 20–29 years old (52.3%). Most of the respondents held a doctoral degree or higher (36.9%), and the most common duration of working experience was less than 5 years (66.2%). The major occupation groups of the respondents comprised anesthetists (36.2%) (Table 1).

**Table 1:** Socio-demographic characteristics of respondents at the, comprehensive specialized hospital operation room

	Frequency	
Variables	Number (*N*)	Percentage (%)
Sex		
Male	108	83.1
Female	22	16.9
Age range of respondents (years)		
20–29	68	52.3
30–39	59	45.4
40–49	3	2.3
Occupation of respondents		
Anesthetist	47	36.2
Surgeon	46	35.4
Nurse	18	13.8
Midwifery	19	14.6
Educational qualification		
Bachelor’s degree	45	34.6
Master’s degree	37	28.5
Doctoral degree and above	48	36.9
Working experience (years)		
1–5	86	66.2
6–11	38	29.2
>11	6	4.6

**Figure 1:**
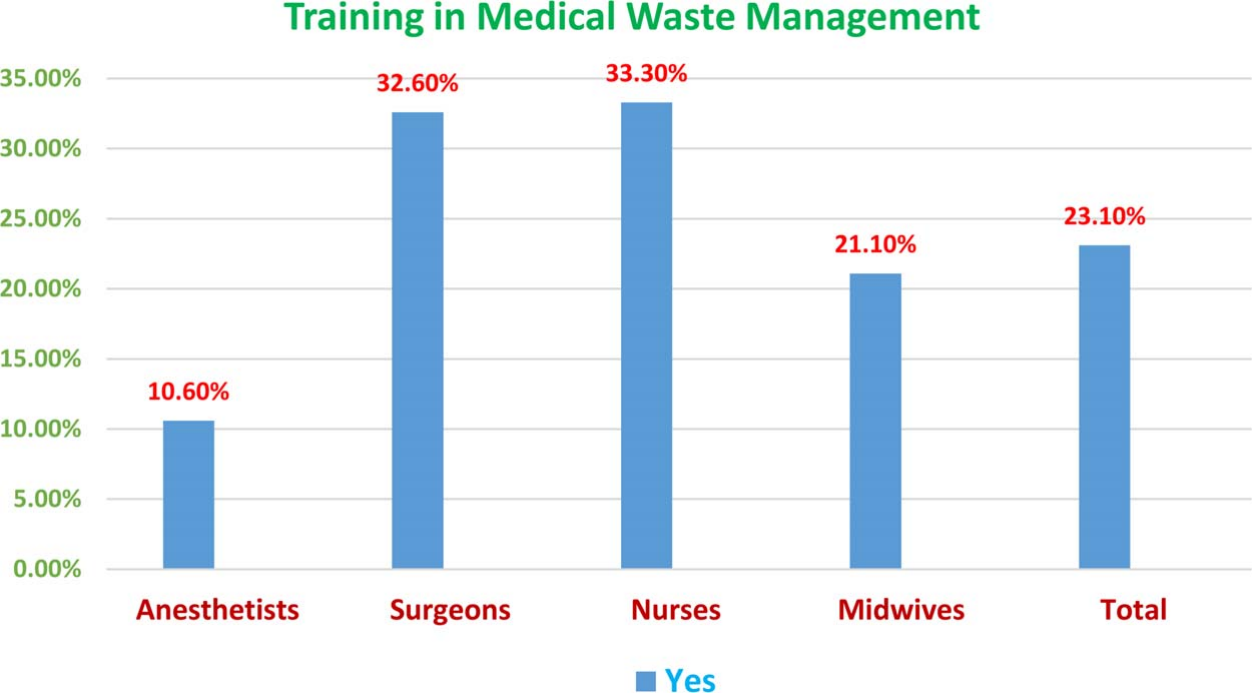
Bar graph of the training status of medical waste management at the comprehensive specialized hospital operation room.

Part B: General information from respondents regarding MWM at the hospital: The general information about MWM given by the respondents is presented in Figure 1. Only 30 (23.1%) of the respondents were involved in training in medical waste management, whereas 100 (76.9%) of the study participants have not participated in training in medical waste. Of those who participated in training in medical waste management, 33.3% were nurses, followed by physicians (32.6%). In terms of medical waste disposal methods at hospitals, many respondents (43.1%) disposed of medical waste in general waste bins; however, many respondents did not know how to dispose of medical waste. Eleven (8.5%) of the study participants dispose of infectious waste themselves, while 7 (5.4%) have medical waste disposal handled by a private infectious waste management company (Fig. 1).

Part C: Knowledge of respondents about MWM: It is generally known that healthcare workers’ knowledge about MWM is fundamental for proper MWM and is the most important aspect of waste disposal methods. The scores relating to the knowledge of the respondents to MWM are summarized in Table 2.

**Table 2:** Level of knowledge of medical waste management at the comprehensive specialized hospital operation room

Knowledge of MWM	‘Anesthetists (*n*=47), *n* (%)	Surgeons (*n*=46), *n* (%)	Nurses (*n*=18), *n* (%)	Midwives (*n*=19), *n* (%)	Total (*n*=130), *n* (%)
Medical west is expired medicine					
Correct	28 (59.6)	33 (71.7)	15 (83.3)	14 (73.7)	90 (69.2)
MW is material contaminated with body fluids					
Correct	40 (85.1)	44 (95.7)	17 (94.4)	16 (84.2)	117 (90.0)
Medical west is vaccine container					
Correct	20 (42.6)	28 (60.9)	11 (61.1)	13 (68.4)	72 (55.4)
Waste generated from healthcare activities is medical waste					
Correct	41 (87.2)	46 (100.0)	18 (100.0)	18 (94.7)	123 (94.6)
Medical waste should not be mixed with general waste					
Correct	41 (87.2)	46 (100.0)	16 (88.9)	16 (84.2)	119 (91.5)
Medical waste should be segregated immediately					
Correct	45 (95.7)	46 (100.0)	14 (77.8)	17 (89.5)	122 (93.8)
The color coding for medical waste is red					
Correct	38 (80.9)	40 (87.0)	14 (77.8)	19 (100.0)	111 (85.4)
The color coding for general waste is black					
Correct	32 (68.1)	39 (84.8)	16 (88.9)	15 (78.9)	102 (78.5)
Liquid medical waste should not be disposed into toilet bowl					
Correct	37 (78.7)	42 (91.3%)	15 (83.3)	12 (63.2)	106 (81.5)
Sharp medical waste should be separated from other wastes					
Correct	45 (95.7)	46 (100.0)	17 (94.4)	19 (100.0)	127 (97.7)
Medical waste should be put into a closed container					
Correct	46 (97.9)	44 (95.7)	16 (88.9)	18 (94.7)	124 (95.4)
Sharp medical waste must be put into a hard container					
Correct 4.3%	45 (95.7)	46 (100.0)	15 (83.3)	17 (89.5)	123 (94.6)
MW container should be filled to no more than three-quarters full					
Correct	37 (78.7)	38 (82.6)	12 (66.7)	16 (84.2)	103 (79.2)
Medical waste container should be sealed every single day					
Correct	33 (70.2)	38 (82.6)	15 (83.3)	14 (73.7)	100 (76.9)
Categorization of the total scores from all 14 items					
Good knowledge	24 (51.1)	39 (84.8)	12 (66.7)	11 (57.9)	86 (66.2)
Moderate knowledge	20 (42.6)	5 (10.9)	4 (22.2)	7 (36.8)	36 (27.7)
Poor knowledge	3 (6.4)	2 (4.3)	2 (11.1)	1 (5.3)	8 (6.2)

MWM, medical waste management.

**Figure 2:**
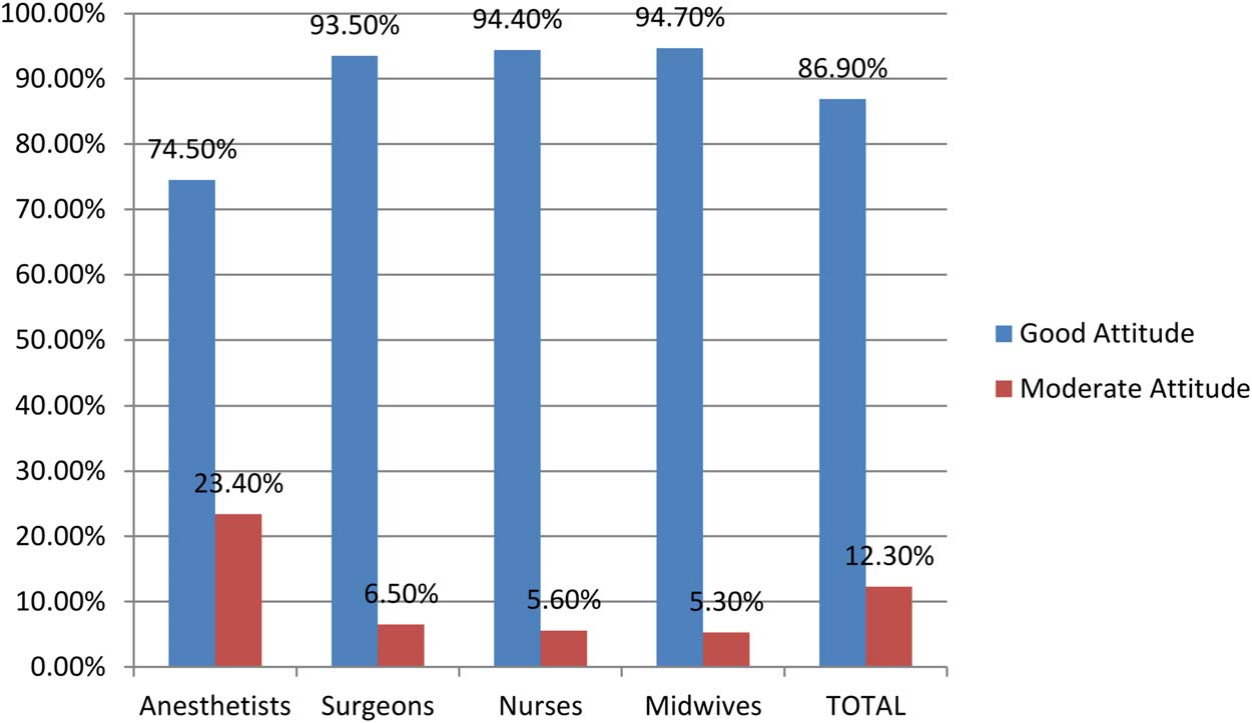
The level of attitude towards medical waste management at the comprehensive specialized hospital operation room.

Part D: Attitude of respondents towards MWM: The scores relating to the attitude of the respondents to MWM are summarized in Figure 2. It was found that 113 (86.9%) of the respondents had a good attitude towards MWM, 16 (12.3%) of the respondents had a moderate attitude, and 1 (0.8%) of the respondents had a poor attitude towards MWM.

**Figure 3:**
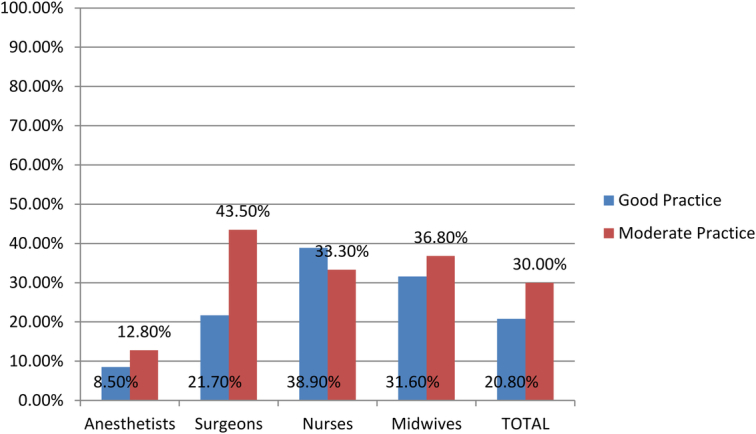
Level of practice in respect of medical waste management at the comprehensive specialized hospital operation room.

Part E: Practice of respondents in Respect of MWM: The respondents’ practice in respect of MWM was determined in this study (Fig. 3). It was found that 27 (20.8%) of the respondents had a good practice towards MWM, 39 (30.0%) of the respondents had a moderate practice towards MWM, and 64 (49.2%) of the respondents had a poor practice towards MWM. Moreover, nurses, midwives, surgeons, and anesthetists had overall good practice scores of 38.9%, 31.6%, 21.7%, and 8.5%, respectively, and surgeons, midwives, nurses, and anesthetists had overall moderate practice scores of 43.5%, 36.8%, 33.3%, and 12.8%, respectively. Anesthetists, surgeons, midwives, and nurses had overall poor practice scores of 78.7%, 34.8%, 31.6%, and 27.8%, respectively.

## Discussion

This study aimed to study the knowledge, attitude, and practices of healthcare workers (HCWs) at a tertiary hospital with University of Comprehensive Specialized Hospital operation room personnel regarding the management of medical waste. In this study, findings showed that there were differences in knowledge, attitudes, and practices of waste management among the study participants. Medical waste management has become a major problem for HCFs worldwide^30,31^. The problem could be aggravated by the lack of adequate KAP and proper waste management utilities. Healthcare workers, particularly waste handlers, are mostly involved in its subsequent management and are potentially at risk^32^. Adequate training is a key factor for effective MWM^33,34^; however, in this study, only 23.1% of the study participants were trained, which is not in compliance with the national and international requirements^35,36^. Prior research has shown that training health workers improves knowledge, attitudes and practices for medical waste management^37,38^.

### Knowledge of waste management

Regarding knowledge of waste management, it was found that 66.2% of the respondents had good knowledge of MWM, around 1/4^th^ of the respondents had moderate knowledge of MWM, and a small number of the respondents had poor knowledge. Our findings were higher in contrast with those reported from a study done in South Africa^39^, which found that only 47.2% of HCWs had a good level of knowledge of the correct disposal of healthcare waste. High scores were recorded by most operation room personnel regarding knowledge of MWM, such as that sharp medical waste should be separated from other wastes (97.7%), medical waste should be put into a closed container (95.4%), waste generated from healthcare activities is medical waste (94.6%), and medical waste should be segregated immediately (93.8%). However, the availability of training and methods of waste disposal scored lowest. About 30 (23.1%) study participants stated that they received some training in MWM, and 56 (43.1%) of the participants took general waste bins. This was much higher than the findings of another study done in India, which reported that only 16.3% of participants had received any training in MWM. However, this was lower than the findings of another study done in Botswana^23^, and Nigeria^40^, which reported that 49.8% and 40% of participants had received any training in MWM, respectively.

### Attitude and practice regarding waste management

Attitude toward waste management among respondents: It was found that most of the respondents (66.2%) had a positive attitude toward medical waste management. This was less than the results of a study in Nigeria^41^ and Ethiopia^42^, where 80% and 78.2% of respondents have a positive attitude towards medical waste management, respectively. This could be due to the low good knowledge scores shown by respondents in this study, which were 23.1%, because training is a key factor for effective medical waste management^33^. This study showed that most of the respondents had a good attitude (86.9%) and a moderate attitude (12.3%) towards medical waste management. This was related to a good level of knowledge and will contribute to a good attitude. In addition, adherence to MWM policies in this study was found to be poor, and HCWs were rarely included in the development of waste-handling policies. There was no evidence that the different departments involved in medical care aimed for proper MWM.

It was found that 27 (20.8%) of the respondents had a good practice towards MWM, 39 (30.0%) of the respondents had a moderate practice towards MWM, and 64 (49.2%) of the respondents had a poor practice towards MWM. Moreover, nurses, midwives, surgeons, and anesthetists all had overall good practice scores of less than 50%. This could be due to the low level of knowledge and training about medical waste management. Most respondents agreed that you should put sharp medical waste into a hard container (76.9%).

### Obstacles to proper medical waste management

The most recognized obstacle to MWM in this study was the “lack of training on the dangers of improper waste management by the HCWs,” with a 23.1% “yes” response. Nurses contributed the highest response, whereas anesthetists had the lowest score. The highest scoring was “nurse staff, with 33.3% responding, and the lowest scoring was “anesthetists’ staff, with 10.6% responding. The highest score in nursing might be explained by the training chance that it will be better than the others.

*Limitation of the study*: Small sample size and tools are not validated specifically for operation room personnel.

*Conclusion and recommendation*: Our study reveals that the majority of study participants have a moderate level of knowledge, a good level of attitude, and poor levels of practice, according to Bloom’s cut-off point. The institution should more fully implement the training for operation room personnel to improve their level of knowledge and practice of medical waste management.

## Ethical approval

An ethical clearance was obtained from the School of Medicine, College of Medicine and Health Sciences, on behalf of the University ethical review board with the number SOM/28/09/2022. “All methods were performed in accordance with the relevant guidelines and regulations”. After the purpose and objective of the study had been informed, Participants were also informed that their participation was voluntary. The data collection tools were anonymous and kept participants’ data in a secure way to maintain the confidentiality of any information provided by participants.

## Consent

Written informed consent was obtained from the patients for publication and any accompanying images. But, currently we have no a copy of the written.

## Source of funding

Not applicable.

## Author contribution

All authors made a significant contribution to the work reported, whether that was in the conception, study design, execution, acquisition of data, analysis, and interpretation, or in all these areas; took part in drafting, revising, or critically reviewing the article; gave final approval of the version to be published; have agreed on the journal to which the article has been submitted; and agree to be accountable for all aspects of the work.

## Conflicts of interest disclosure

The authors declare that the research was conducted in the absence of any commercial or financial relationships that could be construed as a potential conflict of interest.

## Research registration unique identifying number (UIN)

UIN (10082) https://www.researchregistry.com/browse-the-registry#home/.

## Guarantor

Yosef.Belay.

## Data availability statement

The raw data supporting the conclusion of this article will be made available by the authors without undue reservation.

## Provenance and peer review

Not commissioned, externally peer-reviewed.
